# Spatial pattern of foot-and-mouth disease in animals in China, 2010–2016

**DOI:** 10.7717/peerj.4193

**Published:** 2017-12-22

**Authors:** Jun Ma, Jianhua Xiao, Xiang Gao, Boyang Liu, Hao Chen, Hongbin Wang

**Affiliations:** Department of Veterinary Surgery, Northeast Agricultural University, Harbin, China

**Keywords:** FMD, Directional distribution, Spatial autocorrelation

## Abstract

Foot-and-mouth disease (FMD) is a highly contagious disease of cloven-hoofed animals. An outbreak of FMD can produce devastating economic losses for a considerable length of time. In order to investigate the distribution characteristics of FMD in China, data from 2010 to 2016 were collected, including information on 65 outbreaks of FMD (25 by serotype A and 40 by serotype O), and 5,937 diseased animals (1,691 serotype A and 4,284 serotype O cases). Spatial autocorrelation, including global spatial autocorrelation and local spatial autocorrelation, as well as directional distribution analysis, were performed. Global spatial autocorrelation analysis of FMD cases from 2010 to 2016 did not show clustering (*P* > 0.05). In 2013 and 2014, the FMD serotype A hotspots areas were Tibet (*Z* = 3.3236, *P* < 0.001 in 2013; *Z* = 3.2001, *P* < 0.001 in 2014) and Xinjiang provinces (*Z* = 4.2113, *P* < 0.001 in 2013; *Z* = 3.9888, *P* < 0.001 in 2014). The FMD serotype O hotspots areas were: Xinjiang (*Z* = 2.5832, *P* = 0.0098) province in 2010; Tibet (*Z* = 3.8814, *P* < 0.001) and Xinjiang (*Z* = 4.9128, *P* < 0.001) provinces in 2011; and Tibet (*Z* = 3.0838, *P* = 0.0020), Xinjiang (*Z* = 3.8705, *P* < 0.001) and Qinghai (*Z* = 2.8875, *P* = 0.0039) provinces in 2013. The distribution of FMD cases from 2010 to 2016 showed a significant directional trend (northwest-southeast). In conclusion, our findings revealed the spatial patterns of FMD cases, which may provide beneficial information for the prevention and control of FMD.

## Introduction

Foot-and-mouth disease (FMD) is a highly contagious disease of cloven-hoofed animals (Grubman & Baxt, 2004), including cattle, swine, sheep and goats, as well as many species of wild animals (Anderson et al., 1993). The most common presenting symptoms in infected animals are fever, lameness, and vesicles on the tongue, feet, snout, and teats. A fever usually occurs after the vesicular lesions. Severe lesions appear in areas subjected to physical stress or trauma, and viremia can be detected in most infected animals. Depending on the infecting dose and the route of infection, the incubation period ranges from two to 14 days (Gailiunas & Cottral, 1966).

In 1514, the Italian researcher Fracastorius described a disease of cattle with similar symptoms to those of FMD, which is likely the first written description of the disease. Loeffler & Frosch (1897) demonstrated that FMD was caused by a filterable agent. The etiological agent of FMD is the foot-and-mouth virus (FMDV), which belongs to the Aphthovirus genus within the Picornaviridae family (Mumford, 2007). Seven serotypes were serologically identified, including the FMD serotypes A, O, C, Asia 1, South African Territories (SAT) 1, SAT 2, and SAT 3, and each serotype includes multiple subtypes (Bachrach, 1968). FMDV replicates in infected animals and usually spreads to susceptible animals by aerosol, via the respiratory route (Donaldson, 1987). FMDV is excreted into milk, semen, urine, and feces (Donaldson, 1987; Hyde, Blackwell & Callis, 1975).

In adult animals FMD does not produce high mortality, but it results in weight loss, decrease in milk production, loss of draught power, as well as infertility and abortion in dams. In young animals, however, FMD mortality can be high and FMDV can affect heart function (Brooksby, 1982). FMD outbreaks in disease-free countries and regions can cause losses of more than US$1.5 billion per year (Knight-Jones & Rushton, 2013; Leforban, 1999). As a result, FMD affected areas are subject to trade embargoes and cannot export animals or animal products to FMD-free countries.

**Figure 1:**
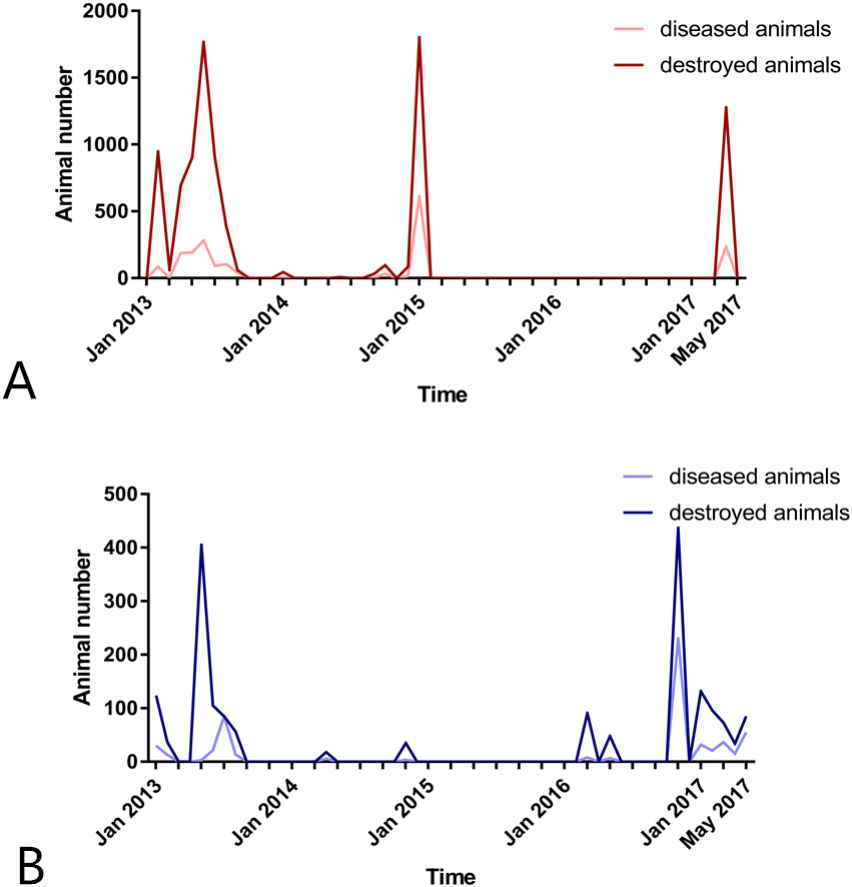
Number of foot-and-mouth disease diseased and sacrificed animals in China between January 2013 and May 2017. (A) Foot-and-mouth disease serotype A; (B) foot-and-mouth disease serotype O.

Through unremitting efforts, several developed countries have eliminated FMD and remain free of the disease, however FMD is still prevalent in many developing countries. In China, recent FMD epidemics have mainly been caused by serotypes A and O, FMD serotype Asia 1 has not been reported since 2009 (Jijun, Jianhong & Xiangtao, 2015). Between January 2013 and May 2017, 44 FMD outbreaks were reported by the Ministry of Agriculture of the People’s Republic of China, 26 of which were caused by serotype A, and 18 by serotype O. The total number of diseased animals during this period was 2485, and 10,939 animals were sacrificed (Figs. 1A and 1B). As shown in Fig. 1, the number of FMD outbreaks reported in 2017 was higher than in 2016, which is a reminder that more effective measures must be implemented to prevent future epidemics.

In this study, we collected data on FMD from 2010 to 2016 in China, including information on 65 FMD outbreaks (25 caused by serotype A and 40 by serotype O viruses) and 5,937 diseased animals (1,691 serotype A and 4,284 serotype O cases). Table 1 showed the incidence rate of FMD by year. To investigate the distribution characteristics of FMD, spatial autocorrelation and directional distribution analysis were performed. This study may provide useful information to FMD hotspot areas and alert at-risk communities about the importance of preventing and controlling FMD outbreaks.

## Materials and Methods

### Data

In this study, data on FMD cases reported between 2010 and 2016 were used. These data, including the number of diseased and sacrificed animals, as well as the geographical location of the epidemic regions, were collected from the Ministry of Agriculture of the People’s Republic of China (http://www.moa.gov.cn/) and the World Organization for Animal Health (OIE) (http://www.oie.int/). Detailed information contained in the reports was compared with published studies and verified by consulting with relevant experts. The infected animal species included pigs, cattle, sheep, and goats. The total number of animals in each province was collected from The National Bureau of Statistics of the People’s Republic of China.

**Table 1:** Incidence rate of foot-and-mouth disease in China from 2010 to 2016.

Year	Serotypes			
	A		O	
	Number of cases	Incidence rate (/10^7^)	Number of cases	Incidence rate (/10^7^)
2010	0	0	2,409	27.76
2011	0	0	847	9.73
2012	0	0	479	5.44
2013	992	11.23	167	1.89
2014	64	0.72	10	0.11
2015	610	6.90	0	0
2016	0	0	245	2.76

### Spatial autocorrelation analysis

The distribution of any phenomenon will present a pattern within a space. The geographic patterns include clustered pattern, dispersed pattern and random pattern. Spatial autocorrelation analysis was used to investigate the presence of a clustering distribution and its type. The incidence rate of FMD was taken as the attribute value for each province.

#### Global spatial autocorrelation

Global spatial autocorrelation was applied to analyze the distribution of FMD cases where all provinces were considered as a whole. Global Moran’s *I* statistic was calculated to evaluate the degree of clustering. In this study, Moran’s *I* measured the spatial autocorrelation of province locations and the incidence rate of FMD and was calculated as follows (Cliff & Ord, 1973):

\begin{eqnarray*}& & \text{Moran' s}~I= \frac{n\sum _{i}\sum _{j}{W}_{ij} \left( {X}_{i}-\overline{X} \right) \left( {X}_{j}-\overline{X} \right) }{\sum _{i}\sum _{j}{W}_{ij}{ \left( {X}_{j}-\overline{X} \right) }^{2}} \end{eqnarray*}

where:

*X*_*i*_ = the incidence rate of FMD in the *i*th province;

$\overline{X}$ = the mean incidence rate of FMD in all of the provinces of China;

*X*_*j*_ = the incidence rate of FMD in the *j*th province;

*W*_*ij*_ = a weight parameter for the pair of provinces *i* and *j* that represents proximity;

*n* = the number of provinces in China.

Global Moran’s *I* ranges from −1 to 1, with 1 corresponding to an extremely clustered distribution and −1 corresponding to an extremely dispersed distribution. When the global Moran’s *I* is close to 0, cases are considered to be a random distribution.

#### Local spatial autocorrelation

Local spatial autocorrelation was applied to explore the distribution mode of cases within a particular province. We used hotspot analysis to investigate the local spatial autocorrelation. Hotspot detection can be useful, even if the global pattern is not clustered. Clusters of cases that randomly occur also have an effect on the spread of an infectious disease (Jeefoo, Tripathi & Souris, 2011).

Provinces with significantly higher incident rate of FMD compared to neighboring provinces were identified as hotspot areas. The local Getis-Ord ${\mathrm{G}}_{i}^{\ast }$ statistics and *Z* value were calculated to test for statistically significant FMD local autocorrelation.

Getis-Ord ${\mathrm{G}}_{i}^{\ast }$ was calculated as follows (Getis & Ord, 2010):

\begin{eqnarray*}& & {G}_{i}^{\ast }= \frac{\sum _{j=1}^{n}{W}_{ij}{X}_{j}-\overline{X}\sum _{j=1}^{n}{W}_{ij}}{S\sqrt{ \frac{n\sum _{j=1}^{n}{W}_{ij}^{2}-{ \left( \sum _{j=1}^{n}{W}_{ij} \right) }^{2}}{n-1} }} \end{eqnarray*}

where:

*X*_*j*_ = the incidence rate of FMD in the *j*th province;

*W*_*ij*_ = a weight parameter for the pair of provinces *i* and *j* that represents proximity;

$\overline{X}$ = the mean incidence rate of FMD in all of the provinces of China;

*n* = the number of provinces in China.

*S* = the standard deviation.

The Getis-Ord ${\mathrm{G}}_{i}^{\ast }$ was calculated to determine the spatial dependence of neighboring observations (Hinman, Blackburn & Curtis, 2006). The statistical significance of the local Getis-Ord ${\mathrm{G}}_{i}^{\ast }$ was given by the *Z* value. If ${\mathrm{G}}_{i}^{\ast }\gt 0$ and *Z* > 1.96, the province would be considered a hotspot area, indicating that FMD cases within this province were spatially clustered with a significance level of 95% (*P* < 0.05). This type of analysis was used to demonstrate the presence of significant local clusters.

### Directional distribution analysis

Directional distribution analysis is most often used to determine whether the distribution of a disease shows a directional trend and to visualize the major areas infected. To analyze directional distribution, standard deviational ellipse was used. This method has been widely applied to study epidemics of infectious diseases (Gesler, 1986; Qiu et al., 2014; Ward et al., 2008). This method generates elliptical polygons by calculating the standard distance of a group of points in both the *X* and *Y* directions. The attributed values for the ellipse polygons include the *X* and *Y* coordinates of the mean center, long and short axes, and ellipse orientation. When the distribution of the disease presents a directional trend, it will be reflected in the analysis of these features. Thus, the ellipse helps us to investigate whether the distribution of points shows a particular direction. The standard deviation to represent the dispersion was 68%. The coordinates of 65 FMD epidemic regions in China infected with 2 viral serotypes from 2010 to 2016 (25 with FMD serotype A and 40 with FMD serotype O) were collected, and the data were analyzed.

### Software

Spatial autocorrelation and directional distribution analyses were performed using ArcGIS 10.2 (ESRI Inc., Redlands, CA, USA).

## Results

### Spatial autocorrelation analysis

#### Global spatial autocorrelation

Table 2 presents the results of the global spatial autocorrelation analysis of FMD cases in China from 2010 to 2016. As shown in the table, all global Moran’s *I* statistics were close to zero, indicating that the spatial distribution of both FMD serotypes A and O cases was random, for all years analyzed (2010–2016).

**Table 2:** Global spatial autocorrelation analysis of foot-and-mouth disease cases in China from 2010 to 2016.

Year	Serotypes					
	A			O		
	Global Moran’s *I*	*Z* score	*P* value	Global Moran’s *I*	*Z* score	*P* value
2010	–	–	–	0.0265	1.2091	0.3749
2011	–	–	–	0.0713	1.1030	0.2518
2012	–	–	–	0.0094	0.8365	0.6307
2013	0.0574	1.3762	0.1928	0.0032	0.4015	0.4168
2014	0.0351	1.7691	0.0763	−0.0367	−0.0689	0.8903
2015	−0.0285	−0.6407	0.9023	–	–	–
2016	–	–	–	−0.0355	−0.5994	0.2754

#### Local spatial autocorrelation

**Figure 2:**
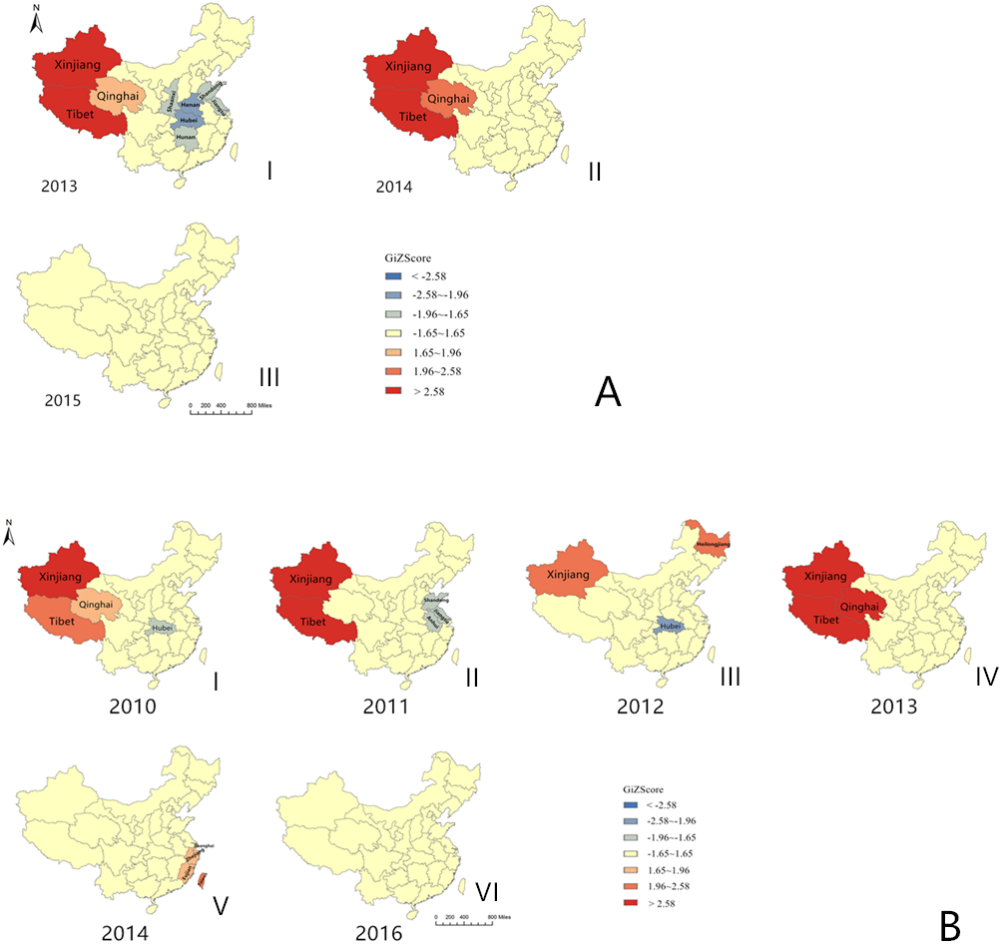
Hotspot analysis of foot-and-mouth disease cases in China from 2010 to 2016. (A) Foot-and-mouth disease serotype A; (B) foot-and-mouth disease serotype O.

Figures 2A and 2B show the results of the hotspot analysis of FMD cases in China. In 2010, Xinjiang (*Z* = 2.5832, *P* = 0.0098) province was a hotspot area for FMD serotype O. In 2011, Xinjiang (*Z* = 4.9128, *P* < 0.001) and Tibet (*Z* = 3.8814, *P* < 0.001) provinces were hotspots for FMD serotype O. In 2012, Hubei (*Z* =  − 2.0016, *P* = 0.0453) province was the cold spot for FMD serotype O. In 2013, Xinjiang (*Z* = 3.8705, *P* < 0.001), Tibet (*Z* = 3.0838, *P* = 0.0020) and Qinghai (*Z* = 2.8875, *P* = 0.0039) provinces were hotspot areas for FMD serotypes O. Xinjiang (*Z* = 4.2113, *P* < 0.001) and Tibet (*Z* = 3.3236, *P* < 0.001) provinces were hotspot areas, whereas Henan (*Z* =  − 1.9692, *P* = 0.0489) and Hubei (*Z* =  − 2.1998, *P* = 0.0278) provinces were cold spots for FMD serotype A. In 2014, Xinjiang (*Z* = 3.9888, *P* < 0.001) and Tibet (*Z* = 3.2001, *P* < 0.001) provinces were hotspot areas for FMD serotype A. In the years 2015 and 2016, local spatial autocorrelation was weak and the distribution of FMD cases showed a random pattern for serotypes A and O (*P* > 0.05).

### Directional distribution analysis

**Figure 3:**
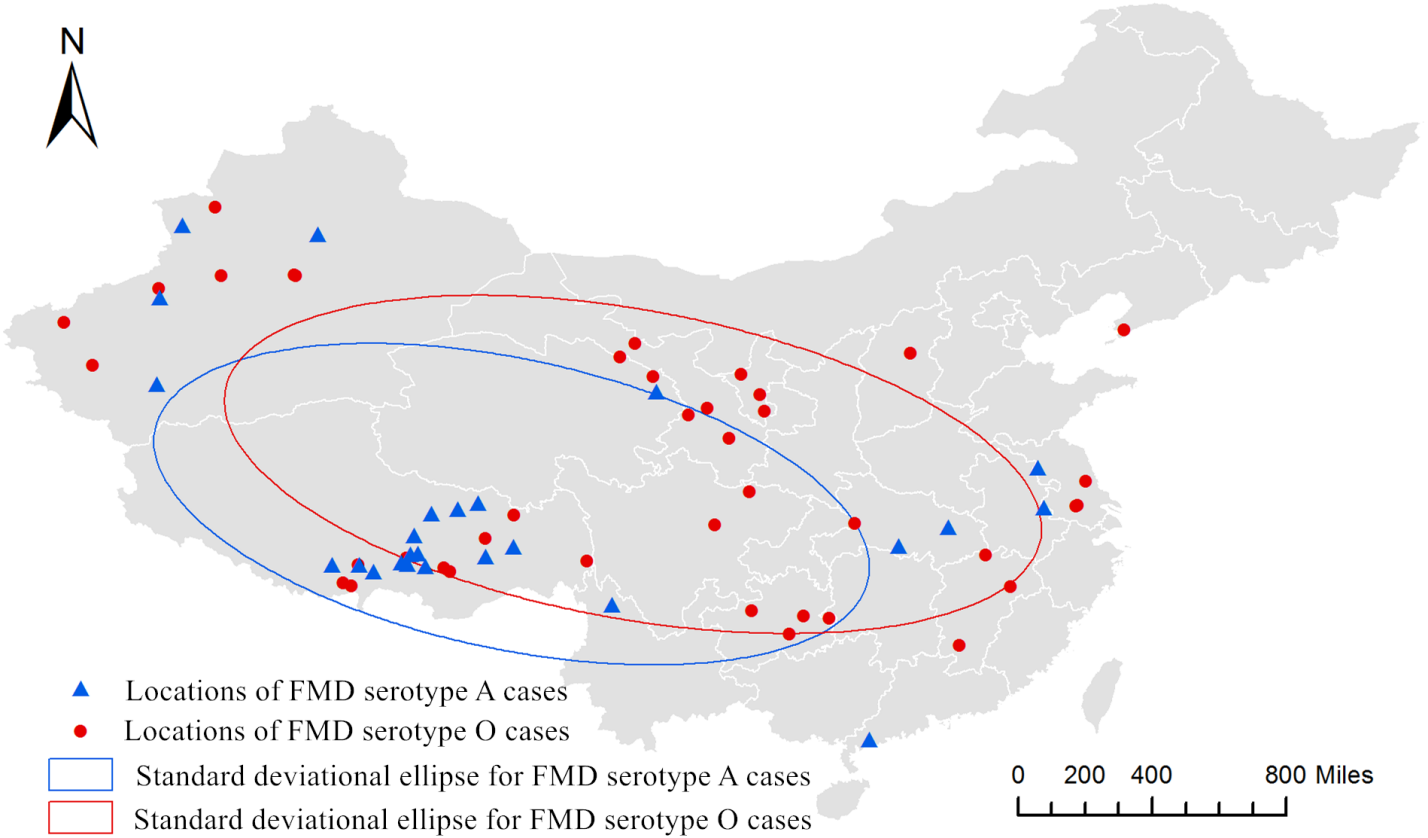
Directional distribution analysis of foot-and-mouth disease serotypes A and O in China from 2010 to 2016.

Figure 3 shows the distribution of epidemic regions caused by FMD serotypes A (*n* = 25) and O (*n* = 40), and the standard deviational ellipses. The attributed values for the standard deviational ellipse are shown in Table 3. As shown in Fig. 3, the distribution of FMD serotypes A and O presented a directional trend and the angular rotations were 101.96° (northwest-southeast) and 100.33° (northwest-southeast). The geographic scope of FMD serotype O was larger than FMD serotype A.

## Discussion

In China, FMD was first reported in 1958 (Bai et al., 2011). In that same year, serotypes O and A isolates were collected from the Xinjiang Uygur Autonomous Region (Bai et al., 2009; Li et al., 2007) and serotype Asia 1 isolate was collected from Yunnan province (Du et al., 2007). In the following years, a series of effective measures were undertaken to prevent the spread of FMD. In January 2012, the OIE and the Food and Agriculture Organization (FAO) jointly developed a global FMD control roadmap, released an FMD global control strategy, and proposed an FMD control objective for the next 15 years (Food and Agriculture Organization of the United Nations (FAO), 2011). As an OIE member, in 2012, China’s State Council released the “National Plan for the Prevention and Control of Animal Diseases in the Medium and Long Term”, putting forward specific objectives to prevent and control FMD serotypes A, O, and Asia 1. In 2014, China submitted the official FMD control plan to the OIE, which was approved in May 2015.

At present, the prevention and control policy that is being implemented in China involves a combination of immunization and culling, and to a large extent the immunity generated by vaccination has effectively prevented and controlled the disease. The FMD vaccines currently used in China mainly include inactivated vaccines and synthetic peptide vaccines. Vaccines developed in China include the swine FMD serotype O Myanmar 98 vaccine (strain Mya 98), the swine FMD serotype O inactivated vaccines (strain OGX09-7 + strain OXJ10-11), and the FMD serotypes A ,O and Asia 1 trivalent inactivated vaccine (strain O/MYA98/BY/2010 + strain Asia 1/JSL/ZK/06 + strain ReA/WH/09). These vaccines are industrially produced and applied in the field (http://sysjk.ivdc.org.cn:8081/cx/). After years of efforts, the prevention and control of FMD in China has largely succeeded. However, due to the existence of FMDV of mixed genetic lineages and different serotypes, the severe and complicated FMD epidemics of neighboring countries, and the increased risk of cross-border importation of epidemic strains, the goal of complete FMD prevention and control in China can currently not be guaranteed.

Spatial autocorrelation has been widely used in epidemic studies (Brooker et al., 2004; Hegazy et al., 2011; Lin et al., 2015; Pope et al., 2007; Ratovonirina et al., 2017), and it is a technique used to detect disease patterns (Moore & Carpenter, 1999). During the time period studied in this report (years 2010–2016), the distribution of FMD serotypes A and O cases showed a random pattern when the study area was considered as a whole. And, as shown in Fig. 3, the diffusion of these cases was saltatory. It is likely that this distribution pattern was caused by the inter-provincial transport of animals. Although the global spatial autocorrelation of FMD cases from 2010 to 2016 did not show clustering, the local spatial autocorrelation analysis did show significant clustering in several years. Clusters of cases that occur randomly also affect the spread of an infectious disease (Jeefoo, Tripathi & Souris, 2011). FMD serotype A cases significantly clustered in Tibet and Xinjiang provinces in 2013 and 2014 (*P* < 0.01), and no local spatial autocorrelation was detected in 2015. FMD serotype O cases significantly clustered in the Xinjiang province in 2010, in Tibet and Xinjiang provinces in 2011, and in Tibet, Xinjiang and Qinghai provinces in 2013 (*P* < 0.01), and no local spatial autocorrelation was detected in 2015. The FMD hotspots concentrated in three provinces (Tibet, Xinjiang and Qinghai) in western China. It is strongly recommended that the government strengthens the prevention and control measures in these hotspots regions, and the export of animals and livestock products should be strictly limited.

**Table 3:** Attributed values for the standard deviational ellipse of foot-and-mouth disease cases in China from 2010 to 2016.

Serotype	Shape area	Center	XStdDist	YStdDist	Rotation
A	309.61	(95.50, 31.90)	15.75	6.26	101.96
O	374.80	(100.73, 33.61)	17.91	6.67	100.33

Directional distribution analysis is commonly used to explore whether epidemic transmission of a disease shows a directional trend (Eryando et al., 2012; Oviedo-Pastrana et al., 2015; Rahmaniati et al., 2014). According to our study, FMD serotypes A and O cases in China showed an obvious distributional trend from 2010 to 2016, and the direction was northwest-southeast. The observed directional distribution of FMD may be related to the transportation of animals from breeding areas. According to the reference laboratory analysis, the FMD serotypes A and O cases that occurred in the Tibet area in 2013 were all distributed along the main traffic highway (Jijun, Jianhong & Xiangtao, 2015). This partly reflects the association that exists between animal transportation and FMD outbreaks. It is also a reminder that the restriction of animal transportation from FMD affected and high risk areas must be strengthened. It is well known that biological, ecological, and meteorological factors can affect the emergence of infectious diseases (Patz et al., 1996). China is vast in territory and there are obvious climate differences in different regions. Thus, meteorological conditions may also influence the transmission of FMDV. The specific factors that explain this pattern of directional transmission need to be further investigated.

We believe that the results of this research are reliable, however there are some limitations. The information collected on FMD cases originated from official sources, and underreporting of FMD cases is likely to exist. If underreporting existed, the spatial autocorrelation would be underestimated.

## Conclusions

The global spatial autocorrelation of FMD cases from 2010 to 2016 in China is not clustered. The hotspots areas for FMD serotype A cases were Tibet and Xinjiang provinces in 2013 and 2014. The hotspots areas for FMD serotype O cases were Xinjiang province in 2010; Tibet and Xinjiang provinces in 2011; and Tibet, Xinjiang and Qinghai provinces in 2013. The distribution of FMD cases from 2010 to 2016 showed a significant directional trend (northwest-southeast).

##  Supplemental Information

10.7717/peerj.4193/supp-1 Supplemental Information 1 Outbreaks of FMD-A in China from 2013 to 2016

10.7717/peerj.4193/supp-2 Supplemental Information 2 Outbreaks of FMD-O in China from 2013 to 2016

## Additional Information and Declarations

### Data Availability

The following information was supplied regarding data availability: The raw data has been provided as Supplemental Files.
